# Dysregulated expression of long noncoding RNAs in gynecologic cancers

**DOI:** 10.1186/s12943-017-0671-2

**Published:** 2017-06-21

**Authors:** Elahe Seyed Hosseini, Matthieu Meryet-Figuiere, Hamed Sabzalipoor, Hamed Haddad Kashani, Hossein Nikzad, Zatollah Asemi

**Affiliations:** 1 Gametogenesis Research Center, Kashan University of Medical Science, Kashan, Iran; 2 Normandie Univ, UNICAEN, INSERM, ANTICIPE U1086 (Interdisciplinary Research for Cancers prevention and treatment, axis BioTICLA (Biology and Innovative Therapeutics for Ovarian Cancer), Caen, France; 3 Department of Nanobiotechnology, Faculty of Biological Sciences, Tarbiat Modares University, Tehran, Iran; 4 Anatomical Sciences Research Center, Kashan University of Medical Sciences, Kashan, Iran; 5 Research Center for Biochemistry and Nutrition in Metabolic Diseases, Kashan, Iran; 6 UNICANCER, Comprehensive Cancer Centre François Baclesse, Caen, France

**Keywords:** Long noncoding RNA, Female reproductive system, Ovarian cancer, Endometrial cancer, Cervical cancer

## Abstract

Cancers of the female reproductive system include ovarian, uterine, vaginal, cervical and vulvar cancers, which are termed gynecologic cancer. The emergence of long noncoding RNAs (lncRNAs), which are believed to play a crucial role in several different biological processes, has made the regulation of gene expression more complex. Although the function of lncRNAs is still rather elusive, their broad involvement in the initiation and progression of various cancers is clear. They are also involved in the pathogenesis of cancers of the female reproductive system. LncRNAs play a critical physiological role in apoptosis, metastasis, invasion, migration and cell proliferation in these cancers. Different expression profiles of lncRNAs have been observed in various types of tumors compared with normal tissues and between malignant and benign tumors. These differential expression patterns may lead to the promotion or suppression of cancer development and tumorigenesis. In the current review, we present the lncRNAs that show a differential expression between cancerous and normal tissues in ovarian, cervical and endometrial cancers, and highlight the associations between lncRNAs and some of the molecular pathways involved in these cancers.

## Background

Several organs of the female reproductive system like the uterus, cervix, ovaries, vagina and vulva are prone to be affected by cancer. The most widespread types of gynecological cancers are uterine cancer of the endometrium, followed by cervical cancer [1]. While ovarian tumors are relatively rare in comparison to other gynecologic tumors and are only the third [2] most common gynecological cancer, they represent the seventh cause of death by all cancer in women worldwide [1]. About 1–2% of all gynecologic malignancies are due to primary carcinoma of the vagina (PCV) which predominantly affects postmenopausal women [3, 4]. Vulvar cancer, which is the twentieth most common cancer in women, is very rare and is associated with HPV infection [4, 5]. Primary fallopian tube carcinoma (PFTC) causes about 0.14%–1.8% of female reproductive malignancies. However, PFTC resembles epithelial ovarian cancer (EOC) in histological and clinical terms [6] and it is thought that at least a subset of ovarian tumors could originate in the Fallopian tube. Therefore, “ovarian cancer” often refers to tumors of both ovarian and Fallopian origin [7, 8].

### Endometrial carcinoma

There are two types of endometrial carcinoma (EC); type I EC and type II EC. These two types are distinguishable on the basis of their pathological and demographic properties. Type I EC, or endometrioid endometrial carcinoma (EEC), develops in hyperplastic endometrial tissue and can affect women both before and after menopause. There is a correlation between type I EC and relatively high levels of circulating estrogen [9]. On the other hand, type II EC, also known as non-Endometrioid Endometrial Carcinoma (NEEC), emerges in the post-menopausal period. Since about 50% of type II tumors recur in the 5 years following surgical resection, their prognosis is poor [10].

### Cervical cancer

Cervical cancer is clinically associated with persistent infection with a ‘high-risk’ subset of human papillomaviruses (HPVs) [11, 12]. There are two subtypes of cervical cancer: squamous cell carcinoma and adenocarcinoma. The thin flat cells that cover the outer part of the cervix lead to squamous cell carcinoma. On the other hand, globular-shaped cells on the inner part of the cervix lead to the less common adenocarcinoma [11, 13]. Screening tests, including conventional cytology, are used to identify pre-cancerous lesions. This method, which is known as the Pap smear, can be utilized for their early detection, thereby helping to optimize therapy [14]. Since there are an estimated 12,990 cases of invasive cervical cancer and 4120 deaths per year worldwide, it is critical to find better biomarkers for an earlier diagnosis to improve treatment efficacy and disease outcome [15].

### Ovarian cancer

The fifth leading cause of cancer death in women in developed countries is ovarian cancer. Since the introduction of platinum-based drugs in clinical practice, no significant improvements in ovarian cancer therapies has taken place over the past four decades [16, 17]. Ovarian cancer is a pathology covering a heterogeneous group of tumors originating in the epithelial cells, germ cells, mesenchyme and Fallopian tube. However, approximately 85–90% of ovarian cancers are of epithelial origin. If the tumor is diagnosed early while it is still limited to a single ovary (Stage I FIGO), five-year survival exceeds 80%. However, owing to the lack of symptoms during the early stages, diagnosis is often delayed until when the disease has already spread to the peritoneal cavity. Therefore, the average 5-year survival for ovarian cancer patients when all stages are considered does not exceed 40% [18]. The two main ovarian cancer diagnostic tests are transvaginal ultrasonography (TVUS) and the measurement of cancer antigen (CA-125) concentration in blood [19], although they lack specificity and sensitivity. The advent of accurate diagnostic tools for ovarian cancer in the early stages and the development of new therapeutic approaches are thus urgently needed to improve the outcome of this pathology.

## Long non-coding RNAs (LncRNAs)

**Fig. 1:**
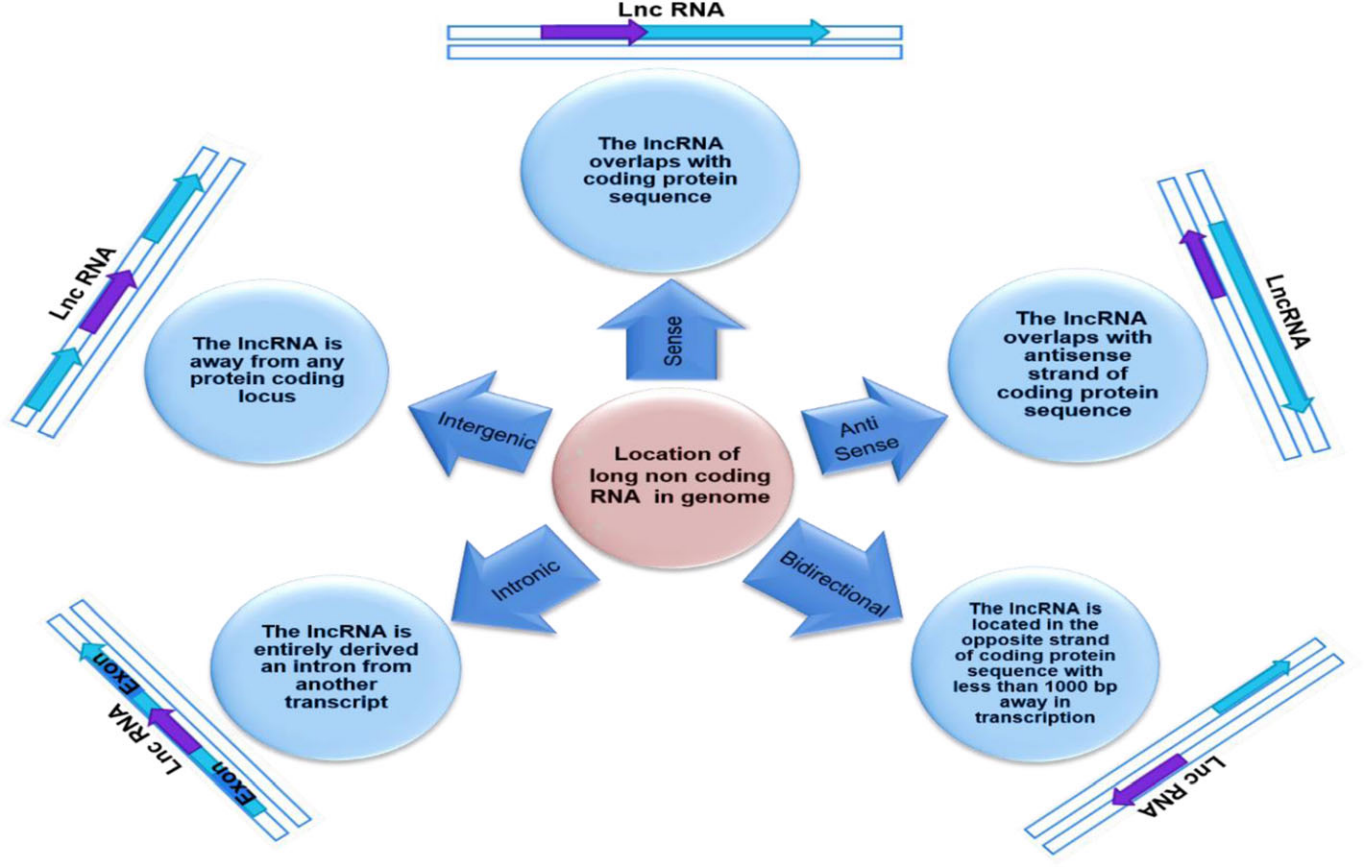
LncRNAs can originate from various genomic locations

Only 2% of the human genome accounts for protein-coding regions, yet more than 70% of the human genome is transcribed in RNA that does not encode proteins [20]. Whether the entirety of the cellular transcriptional output has a functional role is still unknown, and to date only a few ncRNAs have been studied in detail. However, this non-coding part of the genome plays many key roles in the majority of the most critical biological processes such as development, differentiation and the cell cycle, as well as in cancers and other diseases [21]. NcRNAs are generally divided into two classes according to their size [22, 23]. Small ncRNAs are 20–200 nucleotides (nt) in size and long ncRNAs (lncRNAs) are longer than 200 nt, and can exceed 100,000 nt [24, 25]. These lncRNAs can be categorized as exonic, intronic, overlapping or intergenic according to their proximity to the nearest protein-coding transcripts (Fig 1).

## Roles of LncRNAs in cancer

During tumorigenesis, down- or up-regulation of specific lncRNAs occurs relative to the corresponding normal tissues. These lncRNAs thus behave like tumor suppressors or oncogenes [26]. For instance, it has been found that HOTAIR lncRNA overexpression correlates with aggressive breast [27], ovarian [28], cervical [29], endometrial [30], colorectal [31], hepatocellular [32] and gastrointestinal stromal tumors [33], whereas MEG3 lncRNA may act as a tumor suppressor in a variety of human cancers such as ovarian cancer, breast cancer, hepatocarcinoma and uterine cancer [34]. The growth of several cancer cell lines (such as HeLa and MCF7) is inhibited by MEG3 overexpression [35]. Apoptosis in PLC/PRF/5, HepG2, U251 and U87 MG cells is also increased by MEG3 overexpression [34, 36, 37]. A comprehensive overview of lncRNAs in cancer in general was published recently by Evans et al. [38].

### Epigenetic regulation

Cancer can be considered as a disease that is caused by and/or involves aberrant expression of several sets of genes. These modifications include both mutational and epigenetic changes such as methylation, acetylation and phosphorylation of chromatin [39]. Some important cellular genes that are in charge of proliferation, apoptosis and stem cell differentiation undergo epigenetic modifications in cancer [40]. It has been demonstrated that some lncRNAs act by collaborating with Polycomb group repressive complexes (PRC1 and PRC2), which are responsible for the establishment and maintenance of transcriptionally repressive epigenetic modifications in chromatin: trimethylation on lysine 27 of histone 3 (H3K27Me3) and ubiquitinylation on lysine 119 of histone 2A (H2AK119Ub) for PRC2 and PRC1 respectively [41, 42]. Since PRC1 and 2 are believed to be oncogenic drivers in many kinds of cancer [43, 44], this collaboration is especially relevant. For example, the oncogenic lncRNA FAL1 (focally amplified lncRNA on chromosome 1) is in complex with the PRC1 complex subunit BMI1, and this interaction is necessary for FAL1 to exert its oncogenic functions [45]. Because approximately 20% of lncRNAs bind to PRC2 [45], it is thought that this mode of action if one of the main ones. However, it has been shown that PRC2 binds to lncRNAs in a promiscuous way [46]. Several recent publications have reported both specific and non-specific PRC2 binding to lncRNAs. Although lncRNA binding to PRC2 is probably not always specific, numerous cases of PRC2/lncRNA interaction have been shown to be necessary for specific functions [47, 48]. In addition, recent findings suggest that these observations are not mutually exclusive [49].

Epigenetic control of gene expression mediated by lncRNAs also involves transcriptional activation through physical interaction with the MLL complex, which is necessary for the deposition of transcriptionally activating trimethylation on lysine 4 of histone 3 (H3K4Me3) in some instances. An example of such a mode of action is the functional interaction and cooperation between the lncRNA HOTTIP and the MLL complex subunit WDR5 [50].

### Interaction with miRNAs

MiRNAs are an important class of small non-coding RNAs that are involved in several diseases such as cancer [51]. Their main mode of action is through binding to target mRNAs via sequence complementarity, resulting in translation inhibition and mRNA destabilization. Several lncRNAs are capable of binding to miRNAs, thereby preventing them from acting on their target mRNAs. LncRNAs involved in such interactions are termed competing endogenous RNAs (ceRNAs), of which several examples with a role in cancer have been described. For example, MEG3 was recently reported to influence STAT3 expression by altering miR-21 expression in ovarian cancer [34]. A large number of lncRNAs/miRNAs relationships have been reported and/or suggested recently. However, to what extent these interactions are functionally relevant in cells remains a matter of debate [52].

Another relationship existing between lncRNAs and miRNAs is when a lncRNA itself is a precursor RNAs for miRNAs. For example, H19 is a precursor RNA for miR-675. Therefore, the study of the effects of such lncRNA involves taking into account the targets of its derived miRNA [53].

## LncRNAs with a role in several cancers of the female reproductive system

**Fig. 2:**
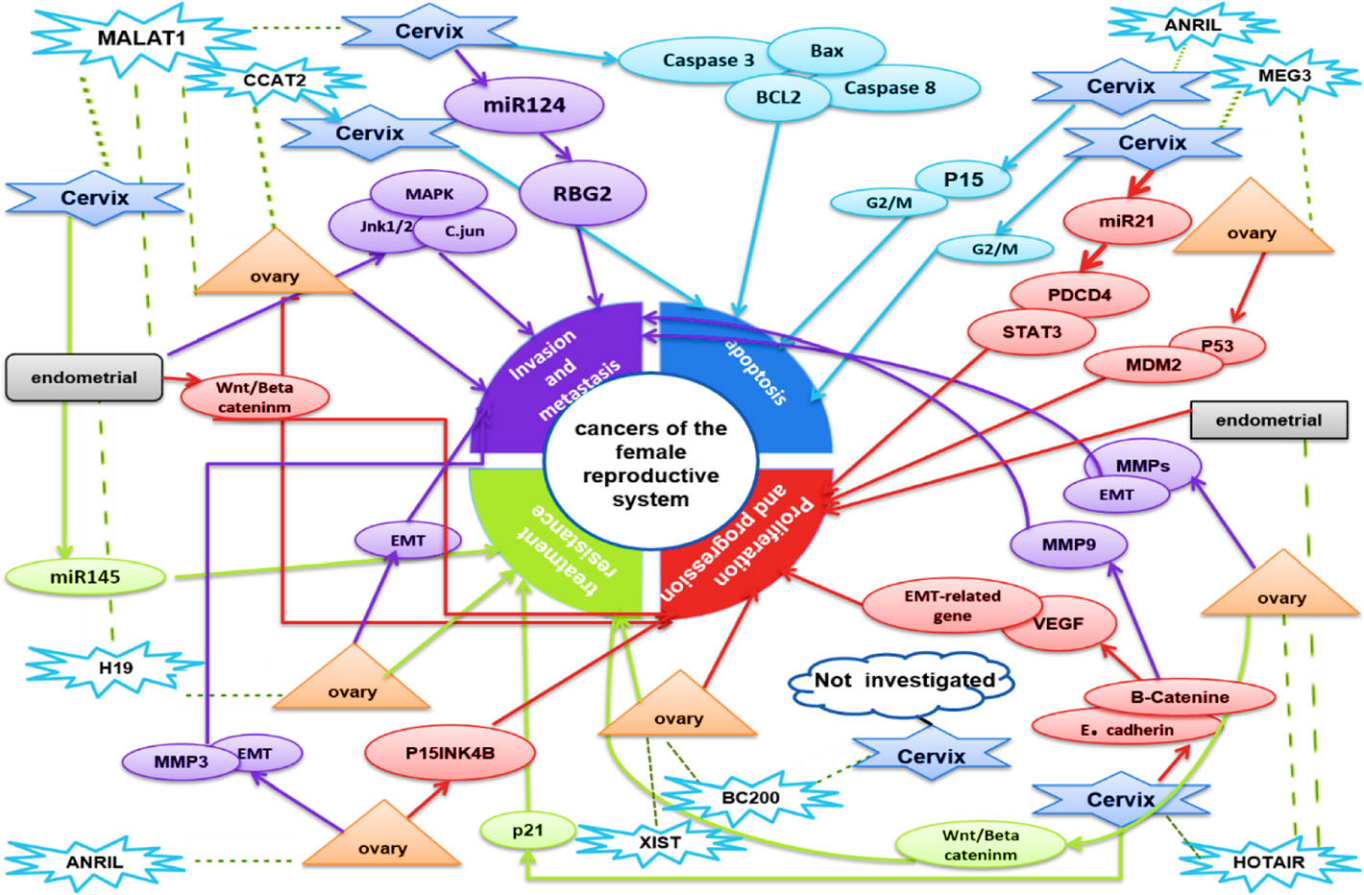
Schematic mechanisms of lncRNA function in reproductive system cancers

Table 1 and Fig. 2 present the roles and functions of lncRNAs, discussed below, that have been studied in several cancers of the female reproductive system.

**Table 1:** LncRNAs with a role in several cancers of the female reproductive system

LncRNA	Size (kb)	Locus	Mechanism	Nature	Related cancer	Ref
MALAT1	8	11q13.1	Strong activation of wnt/beta-catenin signaling pathway through loss of PCDH10 tumor suppressor	Oncogene	Endometrial Carcinoma	[60]
			1- Promotes motility and invasion Increases snail expression; or via MALAT1/miR-124/RBG2 signaling 2-Inhibits cell apoptosis Regulates caspase-3, caspase-8, Bax, Bcl-2, and BclxL 3- Involved in radio-resistance Represses miR-145 reciprocally	Oncogene	Cervical Cancer	[70, 133, 134]
			Correlated with apoptosis and tumorigenicity	Oncogene	Ovarian Cancer	[135]
HOTAIR	2.2	12q13.13	Estradiol-responsive gene induced by estradiol, estrogen receptors and general transcription factors of RNA polymerase II	Oncogene	Endometrial Carcinoma	[27, 75]
			Correlates with migration and invasion	Oncogene	Ovarian Cancer	[28, 78]
			Increases cell migration and invasion	Oncogene	Cervical Cancer	[29, 80], [136]
H19	2.3	11p15.5	Imprinting	Oncogenic	Endometrial Carcinoma	[85–87, 137–139]
			Imprinting	Oncogenic	Cervical Cancer	[140]
			Correlates with migration and invasion	Oncogene	Ovarian Cancer	[89, 141]
MEG3	1.6	14q32.2	Positive regulator of p53	Tumor suppressor	Ovarian Cancer	[34]
			1- Inhibits cell growth Induce G2/M cell cycle arrest and apoptosis 2- Inhibits proliferation and enhances apoptosis. Decreases miR-21-5p levels	Tumor suppressor	Cervical Cancer	[97, 142]
CCAT2	0.34	8q24.21	Correlates with tumor invasion, migration and cell proliferation	Oncogene	Ovarian Cancer	[98]
			Correlates with tumor metastasis	Oncogene	Cervical	[143]
ANRIL	3.8	9p21.3	Correlates with metastasis, cell proliferation both in vitro and in vivo*,* promotion of cell cycle progression and inhibition of apoptosis and senescence	Oncogene	Ovarian Cancer	[102, 144]
			Facilitates proliferation by inhibition of p15	Oncogene	Cervical Cancer	[104]
OVAL	1.4	1q25	Upregulated altogether with p53-regulated genes	Oncogene	Endometrial Carcinoma	[105]
			Not described	Oncogene	Ovarian Cancer	[105]
BC200	0.2	2p21	Translational modulator and correlated with proliferation and chemoresistance	Oncogene	Ovarian Cancer	[121]
			Not studied	Oncogene	Cervical Cancer	[122]
CUDR	2.2	19p13.12	Involved in drug resistance	Oncogene	Cervical Cancer	[111]
			Involved in drug resistance	Oncogene	Ovarian Cancer	[107]

### MALAT1

Metastasis-associated lung adenocarcinoma transcript 1 (MALAT1), also known as NEAT2 (noncoding nuclear-enriched abundant transcript 2), is an 8000 nt-long lncRNA located in chr11q13.1. It is involved in several physiological processes such as epigenetic change of gene expression, nuclear organization and alternative splicing through the modulation of SR splicing factor phosphorylation. It is directly related to a variety of pathological processes ranging from diabetes to cancers [54, 55]. MALAT1 up-regulation has been observed in various types of cancer, where it is associated with tumorigenesis and a decrease in overall survival [56–58].

In endometrioid endometrial cancer, it has been shown that MALAT1 and miR-200c are reciprocally repressed and bound together. When this interaction was altered, EMT was decreased, as well as the invasive capacity of RL-952 cells, leading to a reduced in vivo endometrioid endometrial cancer cell growth in a xenograft model [59].

In addition, high levels of MALAT1 have been reported in endometrioid endometrial cancer [60], in relation with aberrant activation of the wnt/beta-catenin pathway where the wnt-effector transcription factor TCF4 interacts with the MALAT1 promoter region. This wnt/beta-catenin aberrant activation is caused by the loss of expression of the tumor suppressor PCDH10 which normally represses Wnt/beta-catenin activation. Interestingly, PCDH10 is involved in several malignancies such as hepatocellular, colorectal, bladder, nasopharyngeal and cervical cancers, [61–63], while MALAT1 is overexpressed in hepatocellular [64], colorectal [65], bladder [66] and cervical cancer [67]. Whether PDCH10 is involved in MALAT1 overexpression in all these malignancies remains to be determined.

Additionally in cervical cancer, higher levels of MALAT1 are found in cancer tissues compared to normal cervix and are associated with a poor prognosis. MALAT1 is overexpressed in the cervical cancer CaSki cell line and subsequently promotes growth and invasion as well as decreasing apoptosis [68, 69]. MALAT1 is also involved in proliferation in the same cell line, where the expression of cell cycle regulation molecules cyclin D1, cyclin E and CDK6 is decreased following MALAT1 gene knockdown. As a result, cells in the G1 phase are significantly increased [70].

MALAT1 is also overexpressed in the ovarian cancer cell line SKOV3ip, which is derived from SKOV3 with a more metastatic phenotype [71]. Furthermore, MALAT1 inhibition markedly suppresses tumorigenicity in SKOV3 ovarian cancer cells and changes the expression of several genes that are involved in cell proliferation, metastasis and apoptosis. However, the mechanisms by which MALAT1 regulates gene expression in this context is still unclear and requires more detailed evaluation [72].

### HOTAIR

The HOX transcript antisense intergenic RNA (HOTAIR) lncRNA is 2158 nucleotides long and has 6 exons. It is located at the antisense strand of the HOXC gene cluster on chromosome 12q13.13, and was described by Rinn et al. for the first time for its involvement in the determination of the proximal-distal axis during development [47, 73, 74].

HOTAIR gene expression is responsive to estradiol in endometrial carcinoma [75] and when its expression is increased, it leads to increased rates of metastasis and reduced overall survival [30]. Reduction of HOTAIR expression results in a decrease in HEC-1A endometrial cancer cell tumorgenicity and decreases tumor sizes in an in vivo model of xenografted HEC-1A cells [76].

In epithelial ovarian carcinoma, there is a correlation between HOTAIR expression and metastatic stage. The regulation of specific matrix metalloproteinases (MMPs) and EMT-related genes is thought to be responsible for this correlation [77], suggesting that HOTAIR expression levels might constitute a prognostic factor for decreased overall survival [78]. Moreover, in several ovarian cancer cell lines, the expression of HOTAIR causes resistance to cisplatin through wnt/β-catenin pathway activation [79].

In cervical cancer, VEGF and MMP-9 expression are up-regulated by HOTAIR. These two factors play a crucial role in tumor development by increasing migration and invasion. HOTAIR is also correlated with recurrence of cervical cancer. [80, 81].

### H19

H19 is a paternally imprinted gene that encodes a 2300-nt-long lncRNA located in chromosome 11p15.5. It is a bi-functional RNA, acting both as a precursor for miR-675 and a lncRNA [82, 83]. H19 is an oncofetal gene, meaning that it is expressed only in the embryo and not in adult tissue under physiological conditions. However, it is re-expressed in tumors of various origins [84]. It has a maternal expression and its neighboring gene insulin-like growth factor 2 (IGF2) has a paternal allele transcription. Together with IGF2, H19 plays a key role in the normal menstrual cycle and in early pregnancy [85]. Estradiol (E2) has a positive effect on H19 up-regulation in the endometrium. On the other hand, progesterone causes H19 to undergo a down-regulation [54, 85]. H19 induces the expression of a protein superfamily of mitogen-activated protein kinase (MAPK) that includes c-jun, JNK1/2 and the extracellular signal-regulated kinases (ERK) 1 and 2, which are involved in some aspects of the tumorigenic processes in several types of human cancers [86, 87].

Furthermore, H19 has another effect on cell-to-cell adhesion by inhibiting the expression of genes that are involved in this mechanism, including the beta-5, beta-3 and alpha-4 integrins. Thus, the increased motility and invasive potential of some tumor cells might result from this integrin down-regulation [88]. In line with these observations, H19 is overexpressed in ovarian carcinomas, which correlates with the expression of pro-metastatic genes. [89]. In ovarian cancer cells, H19 overexpression enhances migration and invasion [90]. In addition, H19 sequestering of let-7 is required for H19 to function in EMT processes such as cell invasion and migration in ovarian cancer, as well as in uterine serous carcinoma cell lines [91].

H19 expression levels increase throughout endometrial epithelium tumorigenesis. Normal endometrial epithelium has a low level of H19 expression while levels are higher in hyperplastic endometrium. The levels are very high in endometrial carcinoma and even higher during tumor tissue dedifferentiation.

Furthermore, in cervical cancer, markedly increased levels of IGF2 expression and decreased levels of H19 expression have been reported in comparison with those of normal cervical tissues. However, the mechanism promoting this dysregulation is still unclear and needs to be further investigated [92].

### MEG3

Maternally expressed gene (MEG3) is a 1600-nt-long lncRNA located in the locus of DLK1-MEG3 on human chromosome 14q32.3. It was first identified as the ortholog of gene trap locus 2 (*Gtl2*) in mice by Schuster-Gossler [93]. Many normal human tissues express MEG3 and the loss of MEG3 expression has been reported in several types of cancer. MEG3 overexpression can lead to inhibition of proliferation and increased apoptosis in cancerous cells in several ways, either by inducing p53 or in the absence of p53 [94].

MEG3 expression is significantly reduced in ovarian cancer tissue compared to normal ovarian tissue, and its overexpression causes inhibition of growth and proliferation and induces apoptosis in the OVCAR3 ovarian cancer cell line [34, 95]. The other possible mechanism by which MEG3 suppresses tumor growth in this cancer is the RB pathway, in a way that does not depend on p53 in various other cancers such as human pituitary tumors [96].

Unlike normal adjacent tissues, cervical cancer tissues markedly express lower levels of MEG3. Furthermore, MEG3 down-regulation correlates positively with increased tumor size, advanced FIGO stage, metastasis of lymph nodes and HR-HPV positivity. In addition, growth suppression and increased apoptosis of cervical cancer cells is observed after MEG3 upregulation, which demonstrates its tumor suppressive role in this cancer [97].

### CCAT2

Colon cancer-associated transcript 2 (CCAT2) is 1752 nt in size and is located at the 8q24.21 chromosomal region. This lncRNA induces tumor metastasis, progression and chromosomal instability in many different kinds of cancer including gynecologic cancers such as ovary and cervix [98].

Levels of CCAT2 gene expression are higher in tissues and cell lines of ovarian cancer than in corresponding normal tissues. Interestingly, patients who express high levels of CCAT2 have poor prognostic markers such as FIGO stage and distant metastasis. Furthermore, these patients have a much poorer prognosis than those with low CCAT2 gene expression. In addition, in the event of CCAT2 silencing in ovarian cancer cells, cell proliferation, migration and invasion are significantly suppressed [98].

When CCAT2 expression level is inhibited by transfection of siRNA in cervical cancer cells, it leads to significant suppression of their proliferation and survival. There is a correlation between CCAT2 and metastasis which reveals a poor prognosis in patients with cervical cancer [12]. However, the mechanisms mediating the mode of action of CCAT2 in cervical cancer are still unclear [99].

### ANRIL

Antisense non-coding RNA in the INK4 locus (ANRIL) is a 3800-nt-long lncRNA located at the 9p21 chromosomal region. It has been shown that ANRIL regulates its neighbor tumor suppressors CDKN2A/B through epigenetic mechanisms and thereby plays a role in cell proliferation and senescence [100].

ANRIL is considered as an independent prognostic factor in ovarian cancer. By MET and MMP3 modulation, increased migration and invasion results from in vitro ANRIL overexpression in ovarian cancer cell lines. [101]. ANRIL also affects proliferation, which is correlated with the promotion of cell cycle progression and suppression of apoptosis and senescence. The reduced expression of P15INK4B and increased expression of the anti-apoptotic Bcl-2 might explain this phenotype [102, 103].

In cervical cancer cells, the lack of ANRIL expression increases p15 levels but does not influence the expression of p16 or alternative reading frame (ARF) and leads to cell-cycle arrest at the G2/M phase, resulting in suppression of proliferation [104].

### OVAL

Ovarian adenocarcinoma amplified lncRNA (OVAL) is a 1489-nt-long lncRNA whose locus is on chromosome 1q25.3. Genomic evaluation of lncRNAs in high-grade serous ovarian carcinoma (HGS-OvCa) has shown an increase in DNA copy-number of OVAL in 3.9% of ovarian tumors from the TCGA dataset, which is related to an increase in expression of this lncRNA and could be associated with altered p53 activity [105].

OVAL has also been explored in endometrial cancer where type I EC overexpresses OVAL, while it is downregulated in type II EC [106]. Overall, further studies will be needed to characterize the precise roles and function of this lncRNA.

### UCA1a (CUDR)

Cancer up-regulated drug resistant (CUDR) is a 2200-nt-long lncRNA located in the 19p13.1 chromosomal region. Like H19, CUDR is a feto-oncogene expressed only in fetal tissue under normal conditions, except in cardiac tissue [107]. It is re-expressed and up-regulated in cancer tissues from various malignancies including bladder [108] and breast [107]. Some reports suggests that CUDR may play an important role in drug resistance and the transformation of cells through several mechanisms, including caspase 3 down-regulation [109].

CUDR overexpression has been implicated in the resistance of ovarian cancer cells to cisplatin [107] and is associated with a poor prognosis in this malignancy [110].

UCA1 has also been implicated in cervical cancer, where its overexpression promotes resistance of cervical cancer cells to cisplatin [111]. Interestingly, since CUDR is not easily detected in normal tissues and has a low level of baseline expression in comparison with other biomarkers of cancer such as CEA, it might be an effective biomarker to identify the development of cancer and cancer therapeutic responses. However, in order to utilize CUDR expression levels as prognosis tool, further evaluations are required.

### SRA

The steroid receptor RNA activator (SRA) is a 2000-nt-long LncRNA that is located in the 5q31.3 human chromosomal region. It plays a role in regulating the expression of genes induced by steroid receptors. SRA up-regulation has been observed in breast cancer and other tissues which are responsive to steroids, as well as in ovarian cancer.

Regardless of histological tumor grade, endometrial adenocarcinoma tissues consistently express SRA at higher levels than healthy reference tissue samples. This suggests an early role of SRA in the process of tumorigenesis in this tissue. Interestingly, SRA-transgenic mice have been demonstrated to have an elevated rate of apoptosis in order to counteract increased mitotic activity. This suggests that SRA upregulation in tumor tissues could be associated with a loss of its apoptosis-stimulating activities [112].

## LncRNAs with a role in ovarian cancer

Table 2 combines the roles and functions of lncRNAs discussed below that have been studied specifically in ovarian cancer.

**Table 2:** LncRNAs with a role in ovarian cancer

LncRNA	Size (kb)	Locus	Mechanism	Nature	Ref
FAL1	0.566	1q23.3	Correlates with senescence and p21 expression	Oncogene	[45]
ABO73614	1.9	3q24	Correlates with cell proliferation, migration, invasion and promotes apoptosis	Oncogene	[115, 145]
HOST2	1.8	10q23.1	Correlates with proliferation and migration	Oncogene	[116, 146]
LSINCT5	2.43	5q15.33	Correlates with proliferation	Oncogene	[117, 147]
PVT1	1.95	8q24	Correlates with proliferation and apoptosis	Oncogene	[118]
HOXA11-AS	1.62	7p15.2	Correlates with proliferation, migration and invasion	Oncogene	[120]
BC200	0.200	2p21	Correlates with proliferation	Tumor suppressor	[121, 122]

### FAL1

Focally amplified lncRNA on chromosome1 (FAL1) is a 566-nt-long lncRNA and is located on chromosome 1q23.3. Cellular senescence is induced and proliferation rates are hampered in cancer cell lines from various tumoral localizations when FAL1 is knocked down [113]. FAL1 is associated with BMI1, a member of the PRC1 protein complex, and regulates its stability by inhibiting BMI1 degradation. PRC1 can thus repress target gene promoters including tumor suppressor p21. As a result, the cell cycle is dysregulated and tumorigenesis is increased. Furthermore, it has been demonstrated that FAL1 expression is correlated with reduced survival in ovarian cancer patients. Conversely, down-regulation of FAL1 by siRNA delivery in mice xenografted with ovarian carcinoma cells attenuates tumor growth, decreases cell proliferation and increases apoptosis [114].

### AB073614

AB073614 is a 1900-nt-long lncRNA located on chromosome 3q24. It has recently been reported to be overexpressed in ovarian cancer tissues in comparison with adjacent normal tissue. In vitro down-regulation of AB073614 in xenografted mice results in attenuated tumor growth and causes the expression of the proliferation and invasion related proteins PCNA, MMP2 and MMP9 to be decreased. In addition, the ovarian cancer cell lines HO-8910 and OVCAR3 overexpress AB073614 at high levels in vitro*,* and down-regulation of this lncRNA reduces proliferation and results in cell death in these cell lines. Furthermore, in response to down-regulation of AB073614, the expression of the proliferation and invasion related proteins PCNA, MMP2 and MMP9 is reduced in these cells. Also, members of key signaling pathways such as the phosphorylated forms of AKT and ERK are decreased. AB073614 thus seems to play a key role in some critical processes of ovarian cancer, but its precise mechanisms of action have not yet been described [115].

### HOST2

Human ovarian cancer-specific transcript 2 (HOST2) is 1800-nt-long and is located on chromosome 10q23.1. It is the second member of the five human ovarian cancer specific transcripts (HOSTs) that has been reported to be over-expressed in ovarian cancer, and it promotes proliferation and migration in cancer cells as well as tumor progression in xenografted mice. The tumorigenic effects of HOST2 depend on this its ability to behave as a molecular sponge for let-7b, for which it is a direct target, thereby impeding the capacity of let-7b to down-regulate target genes such as myc, hmga2, dicer and imp3 [116].

### LSINCT5

Another lncRNA is human ovarian cancer-specific transcript (LSINCT5) which is 2430 nt long and is situated on chromosome 5q15.33. Many breast and ovarian cancer cell lines as well as tumor sample panels have been reported to overexpress LSINCT5. In addition, knocking down LSINCT5 has been shown to impair cellular proliferation as well as to modify the expression of a number of genes, several of which might play a crucial role in cancer progression. LSINCT5 is considered as a stress-regulated lncRNA and a novel nuclear-expressed gene that might have an important role in cellular proliferation in the development of breast and ovarian cancer [117].

### PVT1

PVT1 is a 2430-nt-long lncRNA located in the 8q24 chromosomal region and neighboring myc, which is amplified in about half of ovarian carcinomas. Guan et al. reported that MYC and PVT1 play independent oncogenic roles in breast and ovarian cancer. However, this idea has recently been challenged by Tseng et al. [118]. The latter group indicated that elevated levels of MYC protein in 8q24-amplified cell lines are dependent on the expression of PVT1 RNA. Furthermore, the correlation of both pvt1 and myc duplication in almost every tumor bearing myc amplification are in favor of this co-dependence. More than 45% of 500 ovarian cancers from TCGA were shown to have amplification of both pvt1 and myc. However, fewer than 1% of cases displayed duplication of only myc or pvt1. The up-regulation of PVT1 in response to carboplatin-docetaxel treatment was recently demonstrated to be a determinant of p53 induction and TIMP1 mRNA expression as well as an associated decrease in cell proliferation. In the same study, tumor progression following down-regulation of PVT1 in tumor xenografts in mice was shown to be increased. This result is in opposition with the study by Tseng et al. who reported an oncogenic role for PVT1, thus underlining the need for further studies on the precise role of the myc/pvt1 association in ovarian cancer [119].

### HOXA11-AS

HOXA11 antisense RNA (HOXA11-AS) is a 1620-nt-long lncRNA located in the 7p15.2 region. It was re-expressed in ovarian cancerous cells but not on the normal ovarian surface epithelium in a cohort study of 18 ovarian cancer patients. Furthermore, proliferation, migration and invasion decreased in the plasmid-based expression of HOXA11-AS in OVCA-433 and C19 ovarian cancer cell lines [80]. The expression of HOXA11-AS was reported to have no effect on the expression of its adjacent genes and no miRNA target site could be predicted, which suggests a possible trans-regulation of distant genes. Therefore, more studies are needed to elucidate its mechanisms [120].

### Bc200

Brain cytoplasmic RNA 1 lncRNA (BCYRN1 or BC200) is 200 nt in size and its locus is on the 2p21 chromosomal region. BC200 expression decreases in ovarian cancer tissues in comparison to adjacent normal tissues, and BC200 suppression has been shown to increase the proliferation of ovarian cancer cells. Interestingly, its expression is induced by carboplatin, thus raising the sensitivity of ovarian cancer cells to this drug [121]. More studies are needed to elucidate its role in modulating sensitivity to cisplatin and its modes of regulation, which could lead to new ways of predicting and/or modulating the clinical response to this drug [122].

## LncRNAs with a role in cervical cancer

Table 3 shows the roles and functions of the lncRNAs that have been studied specifically in cervical cancer.

**Table 3:** LncRNAs with a role in cervical cancer

LncRNA	Size (kb)	Locus	Mechanism	Nature	Ref
LncRNA--EBIC	1.5	12q22	Promotes tumor cell invasion by binding to EZH2 and inhibiting E-cadherin expression	Oncogene	[123]
GAS5	0.651	1q25.1	Hormonal regulation (GR) Tumor	Tumor Suppressor	[127]
lncRNA-CCHE1	2.5	10q21.1	Promotes proliferation by enhancing PCNA	Oncogene	[128]
lncRNA-LET	2.6	15q24.1	Predicts overall survival and serves as a potential therapeutic target	Tumor Suppressor	[129, 148]

### LncRNA—EBIC

EZH2-binding lncRNA in cervical cancer (LncRNA—EBIC) is 1500 nt long and is located in the 12q22 chromosomal region. Only one study has focused on LncRNA—EBIC in cervical cancer while investigating differentially expressed lncRNAs in this pathology. LncRNA-EBIC was found to be able to increase cervical cancer cells invasion through the inhibition of E-cadherin expression. Owing to its association with EZH2, it has been suggested that it could be involved in recruiting the PRC2 complex to genes of interest, but this remains to be formally demonstrated [123].

### GAS5

Growth arrest-specific transcript 5 (GAS5) lncRNA is 651 nt in size and located in the 1q25 chromosomal region [124]. GAS5 is considered to be an lncRNA with tumor-suppressor properties in cancers including breast cancer [125, 126]. Furthermore, it has been reported to be down-regulated in cervical cancer tissues and it also has a marked association with tumor development. In addition, some reports have demonstrated that it is an independent marker to predict clinical outcome in patients with cervical cancer. However, its precise mechanism of action remains to be elucidated by further studies [127].

### CCHE1

Cervical carcinoma high-expressed 1 (CCHE1) lncRNA is 2500 nt in size and is located on chromosome 10. CCHE1 upregulation in cervical cancer is correlated with advanced FIGO stages, larger tumor size, invasion of the uterine corpus, and poor prognosis..CCHE1 binds to PCNA mRNA, increasing its expression and therefore cervical cancer cell proliferation, suggesting that CCHE1 could constitute a prognostic factor for cervical cancer [128].

### lncRNA- LET

A recently identified lncRNA is lncRNA-Low Expression in Tumor (LET), which is 2600 nt in size and is located in the 15q24.1 chromosomal region. It is down-regulated in hepatocellular, gallbladder, esophageal and squamous cell carcinoma as well as in cervical cancer. It has been shown that there is a correlation between expression levels of LET in cervical cancer patients and their clinico-pathological parameters. LET could thus constitute an independent biomarker of prognosis, provided that these observations are confirmed by other studies [129].

## Conclusion

On average, the 5-year survival of cancer patients has been drastically improved over the past decade, although several localizations still represent a major therapeutic challenge. In any case, more effective therapeutic strategies and reliable biomarkers are essential for improving the treatment of cancer.

In the case of gynecologic cancers, both endometrial and cervical cancers are more likely to be diagnosed in the early stages because of their symptoms and the availability of effective screening tools [130]. However, regarding ovarian cancer, late diagnosis owing to asymptomatic development of the disease in the early stages, as well as resistance to existing chemotherapeutic treatments, still pose major difficulties for patient care, and 5-year survival does not exceed 40% all stages considered [131, 132].

The study of lncRNAs in gynecological cancers is still in its infancy, although the last couple of years have seen an increasing number of publications on this topic. Already, the dysregulation of many lncRNAs has been reported in gynecological cancers and has been associated with clinical features, which could prove useful in the future for the design of new biomarkers for prognosis and diagnosis.

However, the exact mechanisms by which lncRNAs exert their action are only seldom studied in details, which underlines the need for more comprehensive studies on their modes of action before even considering using this knowledge to design new therapies or improve existing ones.

Whether some lncRNAs display almost ubiquitous roles in cancer, such as the protein coding p53, or if most of them will show context specific functions will be an interesting question to answer, and might influence the intensity of future efforts made to study the roles of lncRNAs in cancer.

MALAT1 for example is upregulated in many malignancies [55], and at least in ovarian cancer and cervical cancer MALAT1 promotes cell migration and invasion. In addition, MALAT1 is upregulated in EEC through PCDH10 silencing. Since PCDH10 is also silenced in cervical carcinoma, it could be of interest to investigate whether MALAT1 regulation and its effects are somewhat similar in different malignancies [60]. However, some degree of context dependency is to be expected in most cases, since the action of most of the lncRNAs is dependent on other partners, such as protein complexes like PRCs for the epigenetic regulation of gene expression and miRNAs in the case of ceRNA relationships.

The precise understanding of functions and mechanisms of action of lncRNAs may lead to the identification of new vulnerabilities in cancer cells. Direct targeting of lncRNAs through RNA interference is not possible because this technology is not yet available in routine clinical practice. However, once the pathways and proteins responsible for the actions of lncRNAs are identified, it could be possible to target them with specific drugs – possibly even already available drugs, depending on which targets are identified. Therefore, instead of targeting lncRNAs directly, it could be possible, at least in some instances, to simulate their actions in ways that are feasible in clinical practice.

In addition, the use of lncRNAs as biomarkers has triggered considerable excitement in the scientific community, and it can be reasonably expected that in the coming years some lncRNAs might become useful biomarkers for prognosis and for stratifying patients in order to tailor treatment to the needs of individual patients.
